# Bridging Traditional Modeling and Artificial Intelligence in Measles Epidemiology: Methods, Applications, and Future Directions—A Narrative Review

**DOI:** 10.3390/jcm15093242

**Published:** 2026-04-24

**Authors:** Andrei Florentin Baiasu, Alexandra-Daniela Rotaru-Zavaleanu, Ana-Maria Boldea, Mihai-Andrei Ruscu, Mircea-Sebastian Serbanescu, Lucretiu Radu

**Affiliations:** 1 Doctoral School, University of Medicine and Pharmacy of Craiova, 2 Petru Rares Str., 200349 Craiova, Romania; andrei.baiasu@umfcv.ro; 2 Department of Epidemiology, University of Medicine and Pharmacy of Craiova, 2 Petru Rares Str., 200349 Craiova, Romania; alexandra.rotaru@umfcv.ro; 3 Department of Medical Informatics and Biostatistics, University of Medicine and Pharmacy of Craiova, 2 Petru Rares Str., 200349 Craiova, Romania; mircea.serbanescu@umfcv.ro; 4 Department of Hygiene, University of Medicine and Pharmacy of Craiova, 2 Petru Rares Str., 200349 Craiova, Romania; lucretiu.radu@umfcv.ro

**Keywords:** measles, epidemiological modeling, time series analysis, machine learning, seroepidemiology

## Abstract

Measles remains one of the most contagious infectious diseases globally and continues to pose substantial public health risks despite decades of effective vaccination. This narrative review examines both classical and contemporary computational approaches used for measles monitoring, prediction, and control, with particular attention given to the emerging role of artificial intelligence (AI). We synthesized findings from 46 studies; 31 focused directly on measles and 15 on methodologically relevant studies from related infectious diseases (COVID-19, influenza, malaria), selected through searches of PubMed, Scopus, Web of Science, IEEE Xplore, and preprint servers, conducted between June and December 2025. Traditional compartmental models (SIR, SEIR, MSEIR), statistical tools (ARIMA, SARIMA), and seroepidemiological analysis provide transparent, well-characterized frameworks for estimating transmission dynamics and simulating intervention scenarios. Spatial modeling, network analysis, and Monte Carlo simulations have added geographic granularity to outbreak characterization. More recently, AI and machine learning (ML) methods, including supervised algorithms (Random Forest, XGBoost, SVM), deep learning architectures (CNN, LSTM), and hybrid mechanistic ML models, have shown improved predictive performance by integrating multiple data sources: epidemiological records, demographic profiles, mobility patterns, and behavioral indicators. AI-based approaches appear most valuable for high-dimensional risk prediction and image-based diagnostic tasks, while classical models retain clear advantages for policy-oriented scenario analysis. However, no AI-based or hybrid model identified in this review has been adopted into routine national measles surveillance or used for vaccination policy decisions at scale. Important challenges remain: data quality varies across settings, model generalizability cannot be assumed, and computational infrastructure disparities limit deployment in high-burden regions. Explainable AI, federated learning, workforce training for model interpretation, and integration of vaccination registries with mobility and genomic surveillance data represent concrete future directions for strengthening computational support for measles elimination.

## 1. Introduction

The 2023–2024 measles resurgence across Europe, Southeast Asia, and parts of sub-Saharan Africa underscored a persistent vulnerability: even small drops in vaccination coverage can trigger explosive outbreaks of one of the most transmissible human pathogens [1,2]. Measles has a basic reproduction number (R_0_) of 12–18, meaning it spreads through susceptible populations faster than almost any other vaccine-preventable disease [1]. Children, displaced communities, and populations with limited healthcare access continue to bear the heaviest burden, and outbreaks often move too quickly for reactive interventions alone to contain them [2,3].

These dynamics have pushed computational methods to the center of measles epidemiology [4]. Compartmental models, the SIR framework and its extensions (SEIR, MSEIR), have been used for decades to simulate outbreak trajectories based on contact rates, incubation periods, and vaccine effectiveness [5,6]. Their assumptions are well understood, but so are their constraints: they treat populations as homogeneous and struggle to capture spatial or temporal variation, limitations that have long driven interest in alternative analytical tools [6,7].

That interest has intensified over the past several years. Agent-based simulations and geospatial methods can now resolve outbreak dynamics at scales that compartmental models cannot, for example, by showing how spatially clustered vaccine hesitancy creates localized susceptibility that can seed larger outbreaks [8]. Machine learning has also entered the picture. Ensemble approaches combining electronic health records, climatic variables, and web-based signals have improved influenza forecasting, and similar pipelines are being tested for measles surveillance [9]. More broadly, the ability to integrate data from multiple sources, mathematical models alongside machine learning outputs, allows for a more layered view of transmission dynamics [10]. Public health agencies have started incorporating such tools into routine operations, from mapping undervaccinated areas to projecting resource requirements during outbreak response [2,11].

The uptake, however, is uneven. Most applied measles work still uses compartmental models, regression, or time-series analysis. Machine learning applications remain limited to a relatively small number of research groups and are typically confined to specific tasks like case prediction or short-term forecasting. Questions about reproducibility and interpretability are largely unresolved, and very few AI-based tools have been tested prospectively against real outbreak data [4,11].

This narrative review surveys both the established and the emerging approaches. We examine compartmental, time-series, spatial, and seroepidemiological models alongside machine learning, deep learning, agent-based simulation, and data integration methods, assessing performance, interpretability, and maturity for each. Prior reviews have typically addressed these methodological traditions in isolation: compartmental modeling for vaccine-preventable diseases on one hand, AI applications across infectious disease epidemiology on the other. Few have directly compared classical and data-driven methods within the measles context or assessed which approach best fits which public health task. This review addresses that gap. We map classical and AI-based tools to specific operational needs, outbreak prediction, scenario analysis, diagnostic support, resource allocation, and evaluate where machine learning offers genuine analytical advantages over established methods and where it does not. Where we discuss methods developed for influenza or COVID-19, we flag them explicitly as translational possibilities and include them only when the epidemiological characteristics and data structures are sufficiently similar to measles.

We adopted a narrative review framework because the computational methods examined here span fundamentally different analytical paradigms, mechanistic differential equations, frequentist time-series models, supervised and unsupervised machine learning, and hybrid architectures, with incompatible assumptions, validation strategies, and performance metrics. This methodological heterogeneity precludes the standardized quality appraisal and quantitative synthesis required by systematic review protocols, making narrative synthesis the appropriate approach for a comparative methodological assessment of this scope.

## 2. Methods

### 2.1. Literature Search and Source Selection

This narrative review draws on literature identified through searches of PubMed/MEDLINE, Scopus, Web of Science Core Collection, IEEE Xplore, and the preprint servers medRxiv, bioRxiv, and arXiv, conducted between June and December 2025. Google Scholar was used as a supplementary source for gray literature and conference proceedings. We also reviewed reference lists of key articles and consulted institutional publications from WHO, CDC, and ECDC.

Search terms combined measles-related vocabulary (including “rubeola”) with methodological terms using Boolean operators. Representative search strings included: (“measles” OR “rubeola”) AND (“machine learning” OR “deep learning” OR “neural network”); (“measles” OR “rubeola”) AND (“SEIR” OR “SIR” OR “compartmental model”); and (“measles” OR “rubeola”) AND (“ARIMA” OR “time series” OR “forecasting”). Analogous combinations were constructed for spatial analysis (GIS, geospatial), seroepidemiological methods, and Monte Carlo simulation. Search strings were adapted to the syntax requirements of each database. Full-text assessment required English-language availability or a sufficiently detailed English abstract. As this is a narrative review intended to provide methodological breadth rather than exhaustive enumeration, we did not register a protocol or apply systematic review reporting standards (e.g., PRISMA); this approach and its limitations are discussed in Section 2.3.

### 2.2. Scope and Inclusion Rationale

Our aim was to identify and critically discuss the principal computational approaches applied to measles epidemiology, prioritizing breadth of methodological coverage over exhaustive enumeration. We focused on peer-reviewed original research, systematic reviews, and methodological papers published between 2010 and 2025 that reported quantitative results or described a sufficiently detailed analytical framework. Exceptions were made for earlier foundational works, such as Anderson and May’s compartmental modeling, that continue to inform current practice.

We included a limited number of studies from related infectious diseases, influenza, COVID-19, and malaria, when they met transferability criteria: similar epidemiological characteristics, comparable surveillance data structures, and an explicit methodological framework applicable to the measles context. Each such study is clearly identified in our synthesis, and its relevance to measles is discussed rather than assumed.

We excluded editorials and commentaries without original methods, conference abstracts without full-text availability, purely clinical studies without a computational dimension, and studies on unrelated diseases without clear methodological transferability.

### 2.3. Selection Process and Synthesis Approach

Studies were selected iteratively as the review took shape, guided by the eligibility criteria above. Study identification, screening, and final selection were performed by two authors (A.F.B. and A.-D.R.-Z.), with disagreements resolved through discussion and consultation with a third author (A.-M.B.). We did not apply a formal quality appraisal instrument such as ROBINS-I or the Newcastle–Ottawa Scale; instead, methodological rigor was assessed qualitatively during synthesis, with attention to sample size, validation strategy, reproducibility, and transparency of reporting. We acknowledge this as a limitation: the absence of standardized quality scoring may introduce selection bias, likely favoring studies with more detailed methodological reporting. To mitigate this, we prioritized peer-reviewed publications, sought original sources over secondary summaries, and maintained critical appraisal throughout the synthesis, explicitly noting methodological weaknesses in individual studies where relevant (e.g., Section 4.1.1 and Section 4.1.2). A total of 46 studies were examined, where 31 focused directly on measles and 15 on methodologically relevant studies from related infectious diseases.

A comprehensive overview of all 46 included studies is provided in Supplementary Table S1, which details author, country/region, disease focus, study type, model type, data source, sample/time period, key outcomes and performance metrics, and key limitations for each reference. For the non-measles studies included on the basis of methodological transferability, an additional column provides the explicit rationale for their inclusion in the synthesis.

## 3. Classical Dominance and the Emerging Role of AI in Measles Epidemiology

### 3.1. Classical Approaches: Compartmental, Time-Series, Spatial, and Seroepidemiological Models

Compartmental epidemiological models have been central to measles modeling for decades. They divide a population into states, Susceptible (S), Exposed (E), Infected (I), Recovered (R), and use systems of ordinary differential equations to describe transitions between them. The basic SIR and SEIR structures have been extended over time to include vaccinated, hospitalized, and self-isolated compartments, along with age-stratified and spatially stratified variants [12,13]. Recent extensions of compartmental frameworks have introduced fuzzy number parameters to account for uncertainty in field data [14]. Spatial analyses have mapped measles cases and vaccination coverage at subnational level [15], while Sbarra et al. applied a fully calibrated subnational SEIR framework across 79 Ethiopian zones and 24 age groups, integrating vaccination geodata and population mobility estimates; their model fitting via maximum likelihood estimation identified reporting rates below 3% across regions and suggested that vaccine effectiveness values substantially lower than the commonly assumed 93% efficacy yielded better model fits, highlighting the challenges of parameter estimation in settings with incomplete surveillance data [16].

Time-series methods take a different angle: they work directly from observed incidence data without imposing mechanistic assumptions. ARIMA and SARIMA are the most common tools in this category, effective at capturing seasonal patterns and producing short-horizon forecasts. Wudu et al. applied ARIMA(3,1,1) to Ethiopian surveillance data spanning 2018–2022 in East Gojjam zone, selecting the model based on the lowest Bayesian Information Criterion (BIC) among candidates; their forecasts projected a continuing upward trend, with cases expected to rise from 576 in 2023 to 908 by 2027 [17]. The approach works well when reporting is complete and consistent, but its accuracy drops in settings where case notification is delayed or unreliable, precisely the context where measles burden tends to be highest.

Spatial modeling brings in the geographic dimension that other approaches miss. GIS-based methods combine herd immunity estimates from serosurveys with local demographic and environmental variables to identify risk clusters and map subnational variation in outbreak likelihood. Monte Carlo simulation is frequently added to propagate uncertainty through these estimates. GIS-based spatial methods have been more thoroughly validated for other infectious diseases, COVID-19 in particular [18,19,20], rather than for measles directly. Their inclusion here reflects methodological transferability: the spatial analytical frameworks (hotspot detection, geographically weighted regression, mobility-informed risk mapping) are disease-agnostic and have been applied to measles surveillance data in more recent work [15,16], though with less operational validation than in the COVID-19 context. Their use in operational measles surveillance, while expanding, remains less developed.

Seroepidemiological analysis fills a gap that other methods leave open. By measuring measles-specific IgG antibodies across a population, it captures actual immunity levels, which can differ meaningfully from administrative vaccination coverage, especially in settings with cold-chain disruptions or where vaccines are administered earlier than recommended. Monte Carlo simulation helps account for assay variability and demographic differences, producing probability distributions of herd immunity that are more informative for risk assessment than coverage statistics alone. Hayford et al. demonstrated the value of serological verification in Zambia, where a post-campaign serosurvey found rubella seroprevalence of 98.4% among children, significantly higher than the 89.8% reported campaign coverage, while also revealing immunity gaps among young adults aged 16–30 years who were outside the campaign’s target range, underscoring how administrative coverage figures can both underestimate (through underreporting) and fail to capture immunity gaps in non-targeted populations [21]. The main constraint is logistical: population-representative serosurveys are expensive and labor-intensive, and they are rarely conducted often enough to support real-time surveillance.

**Figure 1:**
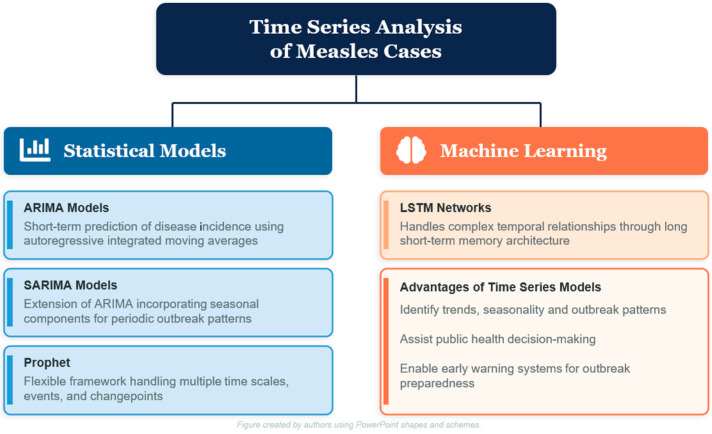
Comparative framework of statistical and machine learning approaches for time-series analysis of measles cases, highlighting established (ARIMA/SARIMA), emerging (tree-based ML), and experimental (deep learning) methods.

**Figure 2:**
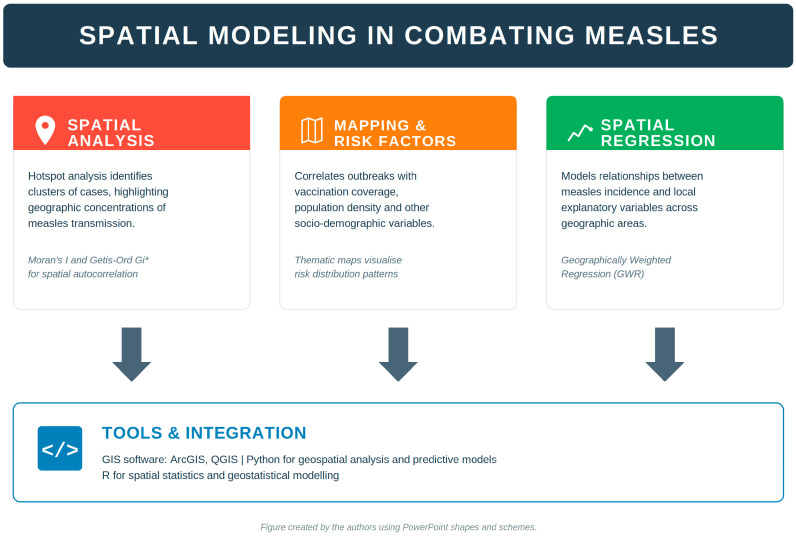
Geospatial Approaches for Measles Surveillance and Intervention Planning, illustrating widely adopted GIS-based approaches currently used in operational measles surveillance. The asterisk in “Getis-Ord Gi*” is part of the standard notation of the statistic and does not denote a footnote.

Each of these approaches has clear strengths and equally clear limitations. Compartmental models offer transparency and support direct policy simulation, but they assume homogeneous populations and depend on accurate parameter estimation. Time-series models capture seasonality effectively but falter when reporting is inconsistent. Spatial methods account for geographic variation but require high-resolution georeferenced data that many settings do not have. Figure 1 provides a comparative overview of the statistical and machine learning approaches discussed in this section and in those that follow, organized along a gradient from established to experimental. Figure 2 illustrates the GIS-based geospatial approaches currently used in operational measles surveillance and intervention planning.

### 3.2. AI and Machine Learning Approaches

AI and deep learning operate differently from the methods described above. They can detect complex, non-linear patterns in large datasets without requiring predefined assumptions about variable relationships, and they can update their predictions as new data become available. The approaches used in measles-related work include tree-based ensemble models, deep neural networks, hybrid mechanistic–ML architectures, and, at the most experimental end, generative models [22,23,24,25,26,27,28,29].

A clear pattern emerges from the reviewed literature: ML adoption in measles epidemiology follows a hierarchy. Tree-based ensemble methods, particularly Random Forest and XGBoost, are the most frequently used. They appear primarily in outbreak risk classification at subnational scales and in vaccination dropout prediction, tasks where their tolerance for mixed data types, missing values, and their built-in variable importance rankings offer practical advantages over classical regression [30,31]. Interpretability is lower than logistic regression but generally sufficient for public health use, especially when supplemented with SHAP value analysis.

Deep learning occupies a more limited role. CNNs have been tested for rash image classification, showing accuracy comparable to or better than clinician assessment under controlled conditions [32], though real-world performance has not been established. LSTMs have been applied to time-series forecasting of measles incidence, with gains over ARIMA most evident in longer, non-linear series [33]. Madden et al. (2024) produced the most rigorous direct comparison available, evaluating deep neural networks against mechanistic models for endemic measles dynamics; their finding that hybrid approaches outperformed either method alone has implications for how the field develops [4]. Graph neural networks (GNNs) have theoretical appeal for modeling spatially structured transmission, but no validated measles application exists to date.

One finding needs to be stated directly: across the 46 studies reviewed, we identified no peer-reviewed evidence that any AI or hybrid model has been integrated into routine national measles surveillance systems or formally adopted for vaccination policy decisions. This observation is consistent with broader assessments of AI readiness in public health: recent systematic reviews have documented that while AI-based early warning systems show promise in research settings, deployment in operational surveillance remains rare, constrained by data infrastructure limitations, workforce capacity gaps, and the absence of regulatory frameworks for algorithmic decision-support tools in public health [34,35,36]. The barriers are not exclusively technical. AI methods require larger and more complete datasets than most passive surveillance systems generate, produce outputs that can be difficult for non-specialist decision-makers to interpret and defend, and raise accountability questions that existing governance structures do not address [37,38].

### 3.3. The Current Landscape: A Methodological Gradient

What the literature shows, overall, is a methodological gradient. Classical models—SEIR-type compartmental frameworks, regression, ARIMA/SARIMA, GIS-supported spatial analysis—remain the default for most applied measles work. The reasons are practical: they match the data that public health agencies collect, they satisfy the interpretability requirements of policymakers, and they fit into established surveillance workflows. AI methods have shown added value mainly in two areas: high-dimensional risk prediction, where tree-based models outperform classical regression, and image-based diagnosis, where CNNs show early promise. For scenario analysis and policy evaluation, classical SEIR and time-series models perform at least as well and are often easier to defend to decision-makers [34].

Table 1 summarizes these comparisons. Two observations stand out. AI methods outperform classical approaches mainly when the task involves high-dimensional inputs or pattern recognition in unstructured data. And the maturity gap between the two is large: established methods rest on decades of operational use, while most AI applications have not moved beyond the emerging or experimental stage. The direction of travel in the field appears to be toward integration, using machine learning to augment specific tasks within a classical analytical framework, not toward replacing compartmental or time-series models wholesale [4,34].

## 4. AI and Machine Learning in Measles Prediction

Section 3 established that AI and ML methods occupy a growing but unevenly adopted position within measles epidemiology. This section examines how specific methods have been applied to specific tasks, organized around the question of what each approach adds, and where it falls short, relative to classical alternatives. The subsections below cover classification and image-based diagnosis, regression and risk estimation, unsupervised clustering, and time-series forecasting and hybrid architectures.

### 4.1. Classification and Image-Based Diagnosis

Classification, assigning observations to discrete outcome categories based on structured or unstructured input features, is the ML task most frequently applied in measles epidemiology. It has been used for two distinct purposes: identifying high-risk areas or individuals from surveillance and demographic data, and automating measles diagnosis from dermatological images. The methods and challenges differ substantially between these two applications.

#### 4.1.1. Surveillance-Based Classification

Gyebi et al. (2023) [30] compared five ML classifiers, Naive Bayes, SVM, ANN, Decision Tree, and Random Forest, against a Generalized Linear Model for predicting confirmed measles cases in Ghana. Their dataset comprised 1797 suspected cases confirmed by laboratory testing, described by six variables: sex, age group, region, settlement type, vaccination status, and measles status. The classes were heavily imbalanced (1696 negative versus 78 positive), and random oversampling was applied to produce 2536 balanced observations. Random Forest achieved the best performance: 92.11% accuracy, sensitivity 0.883, specificity 0.964, PPV 0.963, NPV 0.883, and AUC 0.923, substantially outperforming Decision Tree (78.39%) and GLM (72.87%). Variable importance analysis identified region of residence as the strongest predictor, followed by age group and settlement type [30].

The reliance on random oversampling rather than more robust techniques (SMOTE, class-weighted loss functions) may have inflated these performance estimates. And the modest feature set, six variables, limits the model’s capacity to capture the epidemiological complexity it is credited with modeling. A Random Forest trained on six predictors is not learning subtle non-linear interactions; it is, in effect, a sophisticated decision rule that could likely be approximated by a well-specified logistic model with interaction terms [40,41].

The Ru et al. (2023) [42] framework for identifying U.S. counties at risk of measles outbreaks combined unsupervised clustering (HDBSCAN and unsupervised random forest) with supervised classification, feeding cluster-derived principal coordinates as additional inputs into XGBoost and logistic regression. XGBoost achieved AUC-ROC of 0.920–0.926 and AUC-PR of 0.522–0.532, compared to 0.900–0.908 and 0.485–0.513 for logistic regression. Logistic regression showed higher sensitivity (0.837–0.857 versus 0.704–0.735 for XGBoost), while XGBoost recorded better PPV (0.340–0.367 versus 0.122–0.141) and specificity (0.952–0.958 versus 0.793–0.821) [42]. This hybrid supervised–unsupervised pipeline is one of the few attempts to move beyond purely supervised approaches in measles risk estimation, and it illustrates both the promise and the limitations of the strategy. A PPV below 0.37, even in the best-performing model, means that more than six out of ten counties flagged as high-risk would not experience outbreaks, a false alarm rate that could erode trust if deployed operationally. The authors acknowledged the need for further optimization of unsupervised feature integration [43].

#### 4.1.2. Image-Based Diagnosis

A separate line of work has applied deep learning to automated measles diagnosis from rash images. The clinical rationale is straightforward: measles diagnosis in resource-limited settings often depends on clinical recognition of the characteristic maculopapular rash, and experienced clinicians are not always available [44]. CNN-based systems could, in principle, extend diagnostic capacity. In practice, the gap between controlled evaluation and clinical deployment remains wide.

Glock et al. (2021) developed a ResNet-based measles rash recognition model trained on over 1300 dermatological images, reporting 95.2% classification accuracy, 81.7% sensitivity, and 97.1% specificity [32]. The gap between sensitivity and specificity deserves scrutiny: 81.7% sensitivity means the model misses roughly one in five measles cases. In a public health context where undetected cases can seed transmission chains, this false-negative rate is a meaningful limitation that the authors did not discuss.

Shareef et al. (2024) proposed a YOLOv5-based method trained on the MSID dataset, achieving 92% accuracy with an F_1_ score of 0.92 [45]. The study did not include a direct comparison with clinician assessment, and validation was limited to a single image source. The claim that the model could reduce dependence on human expertise is not supported by the available evidence. Deployment in clinical settings with variable image quality, skin tones, and lesion stages would likely reduce performance.

Naik et al. (2025) introduced a hybrid CNN+RF+KNN framework for measles lesion detection, reporting 99% accuracy [46]. This figure should be interpreted with considerable caution. The evaluation was conducted on a limited dataset without external validation on independent clinical populations. Image-based diagnostic models routinely show performance degradation outside their training conditions; differences in lighting, skin tone, camera quality, and lesion stage can reduce accuracy substantially. Until validated in prospective, multi-site clinical settings, 99% accuracy reflects in-sample performance rather than real-world diagnostic capability.

Across these image-based studies, a common limitation stands out: none has been tested prospectively in the clinical environments where it would actually need to perform. The datasets are small, internally validated, and drawn from controlled sources. This is not unusual for the current stage of medical image classification research, but it means that claims about reducing dependence on clinical expertise are premature [47].

### 4.2. Regression and Risk Estimation

Where classification assigns observations to categories, regression estimates continuous outcomes, case counts, incidence rates, or outbreak trajectories. In measles epidemiology, regression-based ML has been applied to short-term forecasting, quantifying the impact of vaccination campaigns, and assessing how mobility, density, and coverage influence the effective reproduction number [48].

Burtenshaw et al. (2025) [49] proposed a feedforward neural network (FNN) and a biologically informed variant (BINN) for predicting measles outbreak trajectories in the United States, using dynamic time warping for historical feature selection. Over a 34-week testing period, both models achieved mean square errors below 2 (FNN: 1.106; BINN: 1.145), and five-week-ahead predictions aligned with CDC estimates available as of March 2025 [49]. The practical value, accurate short-term forecasting without requiring real-time data collection, is clear. This work remains a preprint, however, and whether these results hold for outbreaks with different dynamics (higher vaccination coverage, different population density) has not been tested.

Kujawski et al. (2024) [31], extending the Ru et al. framework discussed above, used XGBoost with 17 county-level predictor variables to estimate measles risk across the United States. Tested on 2019 data, XGBoost achieved a sensitivity of 0.72, specificity of 0.94, and AUC of 0.92, while logistic regression showed excellent specificity (1.00) and AUC (0.91) but extremely low sensitivity (0.16) [31]. This illustrates a recurrent finding across the studies reviewed here: ML methods outperform logistic regression primarily when maximizing sensitivity is the priority, which is precisely the objective when identifying counties at risk. The authors rightly noted that the optimal sensitivity–specificity tradeoff should reflect local resources and public health priorities.

Alemayehu (2024) [50] analyzed measles vaccination dropout among children aged 12–23 months using Ethiopian Demographic and Health Survey data. Among eight algorithms tested, XGBoost performed best (accuracy 73.9%, AUC 0.813). SHAP analysis identified key dropout predictors: younger maternal age, specific religious affiliations, low parental education, maternal unemployment, residence in Oromia and Somali regions, large family size, and advanced paternal age [50]. An accuracy of 73.9%, while the best among those tested, means that roughly one in four predictions is wrong, a limitation worth acknowledging when considering deployment for resource allocation. The study’s strength lies less in predictive accuracy than in the SHAP-derived variable importance rankings, which offer actionable insight into where and why dropout occurs.

The performance advantage of ML regression over classical alternatives is most pronounced in heterogeneous, multi-variable datasets where non-linear interactions matter. In stable surveillance contexts with well-characterized data structures, simpler regression approaches remain competitive and considerably easier to interpret.

### 4.3. Time-Series Forecasting and Deep Learning Architectures

Deep learning has been explored in measles epidemiology primarily for time-series incidence forecasting in settings where dynamics are complex, non-stationary, or influenced by variables that classical time-series models handle poorly. The architectures of interest are LSTM networks (which learn long-range temporal dependencies), CNNs adapted for sequential data (which offer computational efficiency and consistency), and, more experimentally, graph neural networks, though no GNN application specific to measles was identified in this review [33,51,52,53].

The most rigorous comparative evidence comes from Madden et al. (2024) [4], who systematically evaluated deep neural network models against mechanistic approaches for endemic measles dynamics. Their central finding, that hybrid strategies combining mechanistic and deep learning components yielded better forecasts than either alone, is significant because it suggests that the field’s trajectory should point toward integration rather than replacement [4].

Lara-Benítez et al. (2021) [54] compared eight deep learning architectures across more than 38,000 models applied to over 50,000 time series spanning multiple infectious diseases. LSTM models delivered the most accurate predictions overall, while CNNs achieved comparable accuracy with less variation across configurations and greater computational efficiency [54]. This study evaluated architectures across multiple infectious diseases, not measles exclusively. We include it because the time-series structures and forecasting horizons tested are directly applicable to measles surveillance data, though whether the reported performance advantages transfer to the sparser, more episodic reporting patterns characteristic of measles remains an open question. For measles forecasting, this suggests that LSTMs are preferable when accuracy on complex temporal patterns is the priority, while CNNs offer advantages where computational speed and robustness to hyperparameter choices matter more. Whether these general findings transfer to the specific characteristics of measles surveillance data, which tend to be sparser, more irregular, and more subject to reporting lags than the benchmark series used in this comparison, is an open question and a clear research gap.

### 4.4. Hybrid Mechanistic–ML Architectures

Hybrid approaches that graft deep learning onto the interpretable structure of SEIR/SIR models represent one of the more actively explored directions in the field. The rationale is practical: mechanistic models provide a sound framework for describing transmission processes, but their performance depends on accurate parameter estimation, a task that is difficult when data are incomplete, outdated, or collected under inconsistent protocols [39]. Deep learning can address this by estimating epidemiological parameters in real time from large historical datasets, incorporating contextual factors (mobility, weather, social behavior) that classical formulations typically exclude, and modeling non-linear dynamics that enable counterfactual scenario generation with greater fidelity [55,56].

Aslam et al. (2024) [57] addressed a problem specific to this class of models: catastrophic forgetting in neural networks exposed to incrementally new data. Their Continuous Learning (CEL) model uses Elastic Weight Consolidation to penalize changes to previously learned parameters via the Fisher Information Matrix. Evaluated on influenza, mpox, and measles data, the model showed high R^2^ values and reduced forgetting relative to benchmarks [57]. The reported forgetting rate of 65%, while lower than comparison models, still means that roughly two-thirds of previously learned information is lost when new domains are introduced. The 18% improvement in memory stability over baselines is meaningful but falls well short of what would be needed for reliable deployment in surveillance systems that must maintain accuracy across multiple epidemic waves. Its relevance to this review rests on the continuous learning methodology rather than measles-specific performance, and we note that domain-specific validation on measles surveillance data alone has not been reported.

The Madden et al. findings, discussed above, reinforce the argument for hybrid approaches: mechanistic structure provides interpretability and epidemiological coherence, while deep learning components improve adaptability and forecast accuracy under changing conditions. The practical challenge is that hybrid models inherit the data requirements and opacity of their deep learning components while adding the parameterization demands of their mechanistic components. Whether this added complexity is justified depends on the specific forecasting task and the institutional capacity of the end user [4].

### 4.5. Summary

Across the studies reviewed in this section, AI and machine learning approaches in measles epidemiology deliver their clearest advantages in three areas: surveillance-based risk classification (where tree-based models consistently improve sensitivity over logistic regression), image-based diagnosis (where CNNs show promise but lack prospective clinical validation), and high-dimensional risk stratification (where the ability to integrate heterogeneous predictors adds value). For interpretability, scenario analysis, and policy-oriented decision-making, classical compartmental and time-series models remain the standard tools. No hybrid or AI-only model reviewed here has been validated in a prospective public health setting for measles, and the gap between proof-of-concept performance and operational deployment remains substantial. Closing that gap will require not only technical advances but also institutional changes: integration into existing surveillance workflows, interpretability standards that satisfy non-technical decision-makers, and regulatory frameworks for algorithmic tools in public health [34,37].

## 5. Discussion

AI and deep learning do not consistently outperform classical methods in measles epidemiology. The studies reviewed here make this clear. For short-term incidence forecasting in settings with reliable surveillance, ARIMA/SARIMA and mechanistic SEIR models often match AI methods in accuracy while requiring less data and being easier to explain. For policy simulation and vaccination scenario planning, mechanistic models have no AI-based equivalent in the current literature; no machine learning tool identified here can replicate their capacity for transparent, parameter-driven intervention testing. AI adds value when the data are high-dimensional, heterogeneous, or unstructured: multi-variable outbreak risk classification, image-based rash recognition, spatial risk mapping with dozens of predictor variables. In those contexts, machine learning does things that classical tools cannot. Outside them, it functions as a complement, not a replacement [14,58,59,60].

### 5.1. What AI Can and Cannot Do for Measles—In Practice

The practical applications emerging from the reviewed studies cluster around two domains: surveillance and forecasting on one side, clinical diagnosis on the other. In surveillance, the clearest value lies in predictive risk maps and time-series models. Risk maps built with Random Forest or XGBoost can identify subnational areas at elevated outbreak risk and help target vaccination campaigns, but they depend on georeferenced input data that many high-burden settings in sub-Saharan Africa and South Asia simply do not have [37,61]. Time-series models can flag early warning signals, but their accuracy falls off quickly beyond short forecast horizons, and they perform worst in settings with irregular reporting, the very surveillance systems where early warning matters most. Scenario simulation tools support resource allocation planning, but the mechanistic models best suited for this purpose require parameter estimates that are often unavailable at subnational levels [61].

For clinical diagnosis, CNN-based tools could extend measles case identification to settings with few trained clinicians. But none of the image-based models reviewed here [32,45,46] has performed reliably across diverse clinical populations, skin tones, or imaging conditions. The training datasets were small, internally validated, and drawn from controlled sources. The practical significance of this limitation is substantial: measles rash looks different on darker skin, overlaps with other exanthems, and is typically photographed under field conditions that differ sharply from curated training sets. These tools will need prospective validation in the environments where they would actually be used before their clinical value can be assessed.

Continuous learning models like the CEL framework [57] address a genuine problem of model degradation as epidemiological conditions shift between epidemic waves. But the reported forgetting rate of 65% indicates the problem is far from solved. A surveillance tool that loses two-thirds of what it learned from one outbreak when adapting to the next is not ready for routine deployment.

This review deliberately included 15 studies from related infectious diseases, primarily COVID-19, influenza, and malaria, alongside 31 measles-focused studies. The rationale for this inclusion, detailed in Section 2.2, rests on methodological transferability: the computational frameworks developed for these diseases (spatial modeling pipelines, deep learning architectures for time-series forecasting, federated learning approaches) address analytical challenges that are structurally similar across respiratory and vaccine-preventable diseases. However, transferability cannot be assumed. Measles epidemiology differs from these comparators in several important respects: its episodic incidence pattern creates more extreme class imbalance than continuous-transmission diseases; its dependence on vaccination coverage rather than treatment introduces different predictor structures; and the geographic concentration of measles burden in settings with weaker surveillance infrastructure limits the applicability of models developed on high-quality data from well-resourced systems. Each non-measles study included in this review is identified as such in the relevant Results subsection, and its specific methodological relevance to the measles context is discussed rather than assumed.

#### Practical Guidance for Decision-Makers

For national immunization program managers and public health officials considering computational tools, the methodological gradient described in this review suggests a decision framework matched to task and context. For routine surveillance and short-term forecasting in settings with consistent case reporting, classical time-series methods (ARIMA/SARIMA) remain appropriate first-line tools: they are computationally efficient, interpretable, and can be implemented with standard statistical software. For scenario analysis and vaccination campaign planning, mechanistic SEIR models offer transparent parameter-driven projections that can be communicated to finance ministries and international partners; no AI alternative currently matches their utility for policy simulation. AI and machine learning methods add value primarily in two contexts: subnational risk stratification when high-dimensional predictor data (demographics, coverage gaps, mobility patterns) are available, where tree-based models (Random Forest, XGBoost) can identify priority districts more sensitively than logistic regression; and image-based diagnostic support in settings with limited clinical expertise, pending prospective validation of CNN-based tools.

Implementation requirements differ substantially across this gradient. Classical models can typically be run by epidemiologists with standard training; machine learning approaches require either in-house data science capacity or partnerships with technical institutions. In resource-constrained settings, the marginal gains from AI methods may not justify the infrastructure and expertise investments required, particularly when surveillance data quality is the binding constraint. For countries approaching measles elimination, where case detection sensitivity is paramount, the higher sensitivity of ML classifiers may justify additional investment, but only if interpretability requirements can be met and outputs can be integrated into existing decision workflows.

### 5.2. Barriers to Adoption: What Makes Measles Different

The general barriers to AI adoption in infectious disease epidemiology are well described: data scarcity, limited transferability, the tradeoff between accuracy and interpretability, and unequal access to computational infrastructure [38,62,63,64]. What matters more here is how these barriers play out specifically for measles.

Data availability is the first and most obvious problem, but it has a particular structure for measles. Unlike malaria or tuberculosis, where transmission is more continuous, measles surveillance data are characterized by long stretches of zero or near-zero incidence interrupted by sudden spikes. This episodic pattern creates severe class imbalance for classification models and introduces non-stationarity that complicates time-series forecasting, both more extreme than for diseases with steadier transmission. The 1696-to-78 class ratio in Gyebi et al. [30] is representative, not exceptional. Models trained on such data face a choice between oversampling the minority class (risking inflated accuracy estimates) and accepting lower sensitivity for the outbreaks they are supposed to detect.

Transferability raises a second set of measles-specific issues. Measles epidemiology varies with national vaccination schedules (which differ in timing, dose number, and coverage targets), the age distribution of susceptible populations (which depends on vaccination history and natural immunity patterns), and genotype-specific transmission characteristics that vary across WHO regions. A model built on U.S. county-level data [31], a setting with high baseline coverage and sporadic importation-driven outbreaks, has limited relevance to the endemic transmission dynamics seen in the Democratic Republic of Congo or the large-scale resurgences that have occurred in Romania. No study in this review validated its model externally on data from a different country or region. Retraining on local data cannot fully resolve this because the underlying feature distributions, outcome base rates, and data quality differ at a structural level.

Interpretability matters differently for measles than for many other AI applications in health. National immunization programs do not make decisions in isolation, they justify supplementary immunization activities to finance ministries, report coverage targets to WHO regional verification committees, and coordinate supplies through UNICEF. The reasoning behind any risk estimate must be transparent enough to survive that chain. If an XGBoost model flags a district as high-risk but the program manager cannot explain to a regional health director why that district was prioritized over another, the prediction will not lead to action. This reflects how immunization decisions actually get made in the public sector [38].

### 5.3. Gaps in the Current Evidence

Several evidence gaps stand out as particularly consequential.

The most fundamental is the absence of prospective evaluation. No study identified in this review compared AI and classical models side by side on the same measles surveillance data in an operational setting. Existing performance comparisons are retrospective, often conducted on separate datasets, and cannot demonstrate whether AI methods actually improve public health outcomes. Without prospective studies embedded in functioning surveillance workflows, claims about the operational value of these tools remain unsubstantiated.

The image-based diagnostic studies [32,45,46] share a related limitation: all were evaluated on curated datasets, without testing under field conditions, variable lighting, smartphone cameras, diverse skin tones, or co-occurring skin conditions. The difference between accuracy on a curated dataset and performance in a rural health post is likely large, and no study has quantified it. Given that clinical diagnosis drives case reporting in many low-resource settings, this gap has direct implications for surveillance quality.

A further unaddressed question is how AI tools would integrate into the workflows of national immunization programs. Strong predictive performance is insufficient if a model cannot be embedded in the data systems, reporting structures, and decision processes that agencies rely on. The relevant question is not whether an algorithm can estimate outbreak risk, but whether a district health officer—using available tools and data, under time pressure—can act on that estimate before transmission takes hold.

### 5.4. Research Priorities

Among the directions that could address these gaps, we highlight those that seem both most urgent and most achievable in the near term.

Validation infrastructure comes first. Prospective studies are needed that embed AI tools in functioning surveillance systems, compare their outputs with classical methods on the same data in real time, and track whether the resulting decisions actually improve outbreak detection or vaccination coverage. Such studies are expensive and logistically difficult, but without them, AI will remain a promising adjunct to measles epidemiology without a demonstrated operational role [36,65]. The WHO Measles and Rubella Strategic Framework 2030, which already emphasizes surveillance strengthening, provides a natural institutional platform.

Explainability also requires deliberate investment, and it needs to go beyond post hoc SHAP analysis. Model outputs should be designed to match the decision structures that immunization program managers use: district-level risk rankings with named contributing factors, coverage gap estimates that align with vaccination registry data, and scenario projections formatted for WHO verification committees [66,67].

On data integration, the priorities for measles are relatively clear. Linking vaccination registries with mobility data and serological survey results would address the input constraints that limit most current models. Federated learning could be useful where seroepidemiological or vaccination data sit with different national agencies and direct data sharing is not possible—the WHO European Region’s measles elimination verification process, which requires cross-border data synthesis, is one realistic application. Genomic surveillance of circulating measles genotypes, already routine in many European and American reference laboratories, is an underused data source that could strengthen phylogeographic modeling and outbreak attribution [68].

The methods exist, the data sources are available in many contexts, and the public health need is documented. What remains is the harder work, such as testing these tools under real conditions and incorporating them into the systems where measles prevention decisions are actually made.

Equally important are the practical mechanisms through which validated tools reach end users. Open-source software platforms, such as R-based dashboards, Python libraries with standardized epidemiological interfaces, and containerized model deployment pipelines, can lower the technical barriers that currently restrict AI adoption to well-resourced research institutions. System integration requires that model outputs align with the data formats and reporting structures already used by national surveillance platforms (e.g., DHIS2, EWARS), rather than requiring parallel infrastructure. Workforce training is perhaps the most consequential bottleneck: district-level epidemiologists and immunization program managers need practical competency in interpreting model outputs and understanding their uncertainty bounds, not expertise in machine learning itself. Short-course training programs embedded within WHO or CDC regional capacity-building initiatives, designed around case-based scenarios using locally relevant data, represent a scalable approach to building this interpretive capacity.

### 5.5. Clinical Implications and Translational Relevance

The computational tools reviewed here intersect with clinical practice at several points. CNN-based rash classifiers (Section 4.1.2) represent the most direct clinical application: in primary health centers where experienced clinicians are unavailable, a validated smartphone-based tool could shorten the interval between presentation and case notification. That interval matters because measles post-exposure prophylaxis and isolation are time sensitive. However, as discussed, no image-based model has been tested under field conditions with variable lighting, skin tones, or co-occurring dermatoses.

Short-term incidence forecasts, whether from ARIMA or hybrid architectures, have practical hospital-level implications: anticipating admission surges allows procurement of vitamin A, preparation of isolation capacity, and staffing adjustments in pediatric wards. District-level risk maps linked to facility data could support triage of infection prevention resources.

One application is particularly relevant to European settings, including Romania, where recent resurgences have shown that administrative vaccination coverage poorly predicts actual immunity. Seroepidemiological models that quantify immunity gaps can inform clinical decision-making, for instance, by adjusting the index of suspicion for measles in emergency departments evaluating febrile exanthem, where the differential includes rubella, parvovirus B19, and other exanthems.

These applications share one prerequisite: prospective validation under real operating conditions.

### 5.6. Limitations of This Review

Several limitations of this narrative review warrant explicit discussion. First, the narrative design introduces potential selection bias that a systematic review with a pre-registered protocol would mitigate. Our iterative, purposive approach to study selection, guided by methodological breadth rather than exhaustive enumeration, likely favored studies with more detailed reporting, English-language availability, and publication in indexed journals. Studies from non-English sources, gray literature beyond the institutional reports we consulted, and negative findings (models that failed to outperform baselines) may be underrepresented. The direction of this bias likely inflates reported performance metrics for AI methods, as publication bias favors positive results.

Second, heterogeneity across the reviewed studies limits direct comparison. The 46 included studies differ in geographic context (high-income elimination settings versus endemic low-income settings), outcome definitions (suspected versus confirmed cases), time horizons (short-term forecasting versus long-term projections), and performance metrics reported (some studies report AUC, others accuracy, others RMSE, often without confidence intervals or external validation). Table 1 synthesizes reported performance where available, but readers should interpret cross-study comparisons cautiously; apparent differences in method performance may reflect differences in data quality, outcome definitions, or evaluation protocols rather than true methodological superiority.

Third, we did not conduct meta-analysis or compute pooled effect sizes, which would be inappropriate given this heterogeneity and the absence of standardized reporting across studies. Our conclusions are therefore qualitative: we can identify patterns and suggest where AI methods appear to add value, but we cannot quantify the magnitude of improvement with precision.

Finally, the rapid evolution of both AI methods and measles epidemiology means that studies published after our search period (December 2025) may alter the landscape we describe. The field would benefit from a formal systematic review with meta-analysis once reporting standards for AI in infectious disease epidemiology become more established.

## 6. Conclusions

This review covered 46 studies that applied classical and AI-based computational methods to measles epidemiology. The main conclusion is that AI and machine learning extend the analytical toolkit for specific tasks, high-dimensional risk classification, image-based rash diagnosis, and adaptive forecasting in data-rich contexts, but do not displace classical methods. Mechanistic compartmental models and time-series approaches remain the operational standard for scenario analysis, policy simulation, and routine surveillance. No AI-based model identified in this review has entered routine national practice or influenced vaccination policy at scale.

The evidence base supports this at multiple levels. Tree-based ensemble methods (Random Forest, XGBoost) improve on logistic regression for outbreak risk classification, particularly in sensitivity and AUC, but with reduced interpretability and higher data requirements. Deep learning tools, CNNs for rash diagnosis, LSTMs for time-series forecasting, and hybrid mechanistic–neural architectures remain at the proof-of-concept stage. The accuracy figures reported (up to 99% for image classification) come from limited, curated datasets with no prospective validation in clinical or field settings. Hybrid models combining mechanistic structure with data-driven flexibility are the most promising direction for adaptive forecasting, but none have been evaluated under operational conditions.

Moving the field forward requires progress on several fronts. Prospective validation is the most critical: AI tools need to be tested within functioning surveillance systems, compared against classical methods on identical data, and evaluated on whether they improve actual public health outcomes, not just retrospective metrics. External validation across countries and epidemiological contexts is equally necessary; no model reviewed here has been tested outside its training setting, and generalizability cannot be assumed. Designing for explainability from the start is also essential; public health agencies base their resource allocation on risk estimates they can scrutinize and defend, and models that lack transparency will not be adopted regardless of their accuracy.

On a practical level, near-term opportunities exist within current institutional structures. Federated learning could support cross-border model training for measles elimination verification without requiring countries to share raw surveillance data. Connecting vaccination registries with mobility data and serological survey results would address the data limitations that constrain most current models. Genomic surveillance of measles genotypes, already operational in many reference laboratories, could improve outbreak attribution and phylogeographic analysis.

The core challenge remains the distance between controlled performance in research settings and demonstrated value in public health operations. Closing that gap will require not only better methods but also investment in data infrastructure, technical capacity in high-burden countries, and the institutional confidence that follows only from evidence generated under real conditions. Until that evidence accumulates, measles control will depend primarily on classical tools, with AI contributing where and when its value has been shown to hold in practice.

## Figures and Tables

**Table 1:** Comparative performance, maturity, and use cases of computational methods in measles epidemiology.

Method	Measles Task	Comparator	Reported Performance *	Interpretability	Maturity Level	References
SEIR	Transmission dynamics, scenario analysis	–	Baseline; good qualitative fit to outbreaks	High	Established	[5]
ARIMA	Short-term incidence forecasting	–	Baseline RMSE/MAE; competitive for short horizons	High	Established	[17]
XGBoost	County-level outbreak risk	Logistic regression	↑ AUC (≈5–15%), ↑ sensitivity	Medium	Emerging	[31]
Random Forest	Case/risk classification	GLM, Decision Trees	↑ accuracy, ↑ PPV (≈3–10%)	Medium	Emerging	[30]
CNN	Rash image diagnosis	Clinician assessment	Comparable or ↑ accuracy in controlled datasets	Low	Experimental	[32]
LSTM	Time-series forecasting	ARIMA	Context-dependent; gains in non-linear, long series	Low	Experimental	[33]
Hybrid SEIR–DL	Scenario modeling, adaptive forecasting	SEIR	↑ adaptability; better fit under changing dynamics	Medium	Experimental	[4,39]

* Performance metrics are drawn from individual studies with heterogeneous designs, datasets, and evaluation protocols. Direct cross-study comparison of these figures is limited. Arrows (↑) indicate the direction of reported improvement relative to the stated comparator, not standardized effect sizes. Note: No AI-based or hybrid model identified in this review has yet replaced classical models in routine national measles surveillance or vaccination policy decision-making.

## Data Availability

No new data were created or analyzed in this study.

## Supplementary Materials

The following supporting information can be downloaded at: https://www.mdpi.com/article/10.3390/jcm15093242/s1, Supplementary Table S1: Characteristics of Studies Included in the Narrative Review.

## Abbreviations

The following abbreviations are used in this manuscript:

AI	Artificial Intelligence
ANN	Artificial Neural Network
ARIMA	Autoregressive Integrated Moving Average
AUC	Area Under the Curve
AUC-PR	Area Under the Precision–Recall Curve
AUC-ROC	Area Under the Receiver Operating Characteristic Curve
BINN	Biologically Informed Neural Network
CDC	Centers for Disease Control and Prevention
CEL	Continuous Learning
CNN	Convolutional Neural Network
DBSCAN	Density-Based Spatial Clustering of Applications with Noise
DL	Deep Learning
DT	Decision Tree
DTW	Dynamic Time Warping
EWC	Elastic Weight Consolidation
F_1_ score	F_1_ Statistical Measure
F_2_ score	F-beta Score (β = 2)
FNN	Feedforward Neural Network
FPR	False Positive Rate
MSEIR	Maternally immune–Susceptible–Exposed–Infected–Recovered
GNN	Graph Neural Network
GIS	Geographic Information System
GLM	Generalized Linear Model
GPU	Graphics Processing Unit
HDBSCAN	Hierarchical Density-Based Spatial Clustering of Applications with Noise
KNN	K-Nearest Neighbors
LSTM	Long Short-Term Memory
M	Measles-specific compartment (in MSEIR)
ML	Machine Learning
MSE	Mean Squared Error
MSID	Measles Skin Image Dataset
NB	Naïve Bayes
NPV	Negative Predictive Value
PCoA	Principal Coordinates Analysis
PPV	Positive Predictive Value
RF	Random Forest
RNN	Recurrent Neural Network
ROC	Receiver Operating Characteristic
R_0_	Basic Reproduction Number
SARIMA	Seasonal Autoregressive Integrated Moving Average
SEIR	Susceptible–Exposed–Infected–Recovered
SHAP	SHapley Additive exPlanations
SIR	Susceptible–Infected–Recovered
SVM	Support Vector Machine
TPR	True Positive Rate
TPU	Tensor Processing Unit
uRF	Unsupervised Random Forest
WHO	World Health Organization
XGBoost	Extreme Gradient Boosting
