# Supplementary material for: Bridging Traditional Modeling and Artificial Intelligence in Measles Epidemiology: Methods, Applications, and Future Directions—A Narrative Review
Source: J Clin Med. 2026 Apr 24;15(9):3242. doi: 10.3390/jcm15093242 (PMC13163489; doi:10.3390/jcm15093242)
Supplement: Supplementary file 1 [file jcm-15-03242-s001.zip › jcm-4241571-supplementary.pdf]

Supplementary Table S1. Characteristics of Studies Included in the Narrative Review

Ref #	Author/Year	Country/Region	Disease Focus	Study Type	Model Type	Data Source	Sample/Time Period	Key Outcomes/Performance Metrics	Key Limitations	Transferability Justification
1	Tahir et al., 2024	USA	Measles	R	Epidemiological review	CDC surveillance data	2024 outbreak	Described 2024 USA measles resurgence patterns and determinants	Descriptive; no predictive modeling	—
2	Branda et al., 2024	Global	Measles	R	Surveillance review	WHO global surveillance data	2023–2024	Documented global measles trends and public health response challenges	Review; no original analysis	—
3	Sbarra et al., 2023	Global	Measles	C/D	Conceptual framework (expert consultation)	Expert consultation + literature review	N/A	Identified population-level risk factors for measles case fatality through expert-derived conceptual framework	Conceptual; not an empirical model	—
4	Madden et al., 2024	England/Wales	Measles	OR	Hybrid DNN + TSIR	Historical endemic measles data	1944–1965	Hybrid models outperformed standalone DNN or TSIR; lower RMSE with combined approach	Retrospective validation only; no prospective testing	—
5	Alnafisah & Sohaly, 2025	Theoretical	Measles	OR	Markovian SEIR	Simulated data	N/A (theoretical)	Demonstrated stochastic SEIR framework incorporating Markov transitions for measles dynamics	No empirical validation; theoretical only	—
6	Thompson, 2016	Global	Measles/Rubella	R	Dynamic transmission models (review)	Literature review	Historical	Reviewed evolution of measles/rubella dynamic transmission models for policy analysis	Review article; no original modeling	—
7	Amaral et al., 2023	Theoretical	Infectious diseases (general)	OR	Spatio-temporal compartment + point process	Simulated data	N/A (simulation)	Integrated compartmental and point process models for spatio-temporal disease dynamics	Methodological; limited empirical application	Spatio-temporal integration methodology applicable to measles

Supplementary Table S1. Characteristics of Studies Included in the Narrative Review

										outbreak characterization
8	Alvarez-Zuzek et al., 2022	USA (simulation)	Measles	OR	Agent-based spatial model	Simulated populations	N/A (simulation)	Spatially clustered vaccine hesitancy creates localized susceptibility pockets that seed larger outbreaks	Simulation-based; not validated against real outbreak data	—
9	Poirier et al., 2021	France	Influenza	OR	ML ensemble (EHR + web + climate)	EHR, web search, climate data	2014–2019	Improved regional influenza forecasting through multi-source data integration	Influenza-specific; French healthcare system context	Multi-source data integration pipeline applicable to measles surveillance systems
10	Golumbeanu et al., 2022	Sub-Saharan Africa	Malaria	C/D	Mathematical models + ML framework	Malaria intervention data	Various	Demonstrated framework for integrating ML with mechanistic disease models	Malaria-specific parameters; conceptual framework	ML–mechanistic integration framework applicable to measles modeling pipelines
11	Hotez et al., 2020	Global	Measles	C/D	Commentary	Literature review	N/A	Discussed vaccine hesitancy as a social determinant driving measles outbreaks	Conceptual commentary; not empirical	—
12	Branda et al., 2025	Italy (simulation)	Measles	OR	Network-based simulation (SIR)	Simulated network data	N/A (simulation)	Demonstrated network-based computational modeling of measles transmission dynamics	Simulation only; no real-world validation	—
13	Tang et al., 2020	Global	Infectious diseases (general)	R	Multi-compartment models (review)	Literature review	N/A	Comprehensive review of SIR/SEIR extensions and multi-compartment model variants	Review article; no original analysis	Compartmental modeling taxonomy directly applicable to measles

Supplementary Table S1. Characteristics of Studies Included in the Narrative Review

										model selection
14	Dayan et al., 2023	Theoretical	Measles	OR	SEIR with fuzzy number parameters	Simulated data	N/A (theoretical)	Demonstrated fuzzy parameter approach to handle uncertainty in measles transmission estimates	Theoretical; no empirical validation with real data	—
15	Muse et al., 2025	Ethiopia (Somali region)	Measles	OR	Spatial analysis (GIS)	Surveillance and vaccination coverage data	Not specified	Mapped measles cases and vaccination coverage at subnational level; identified spatial clusters	Descriptive spatial analysis; no predictive modeling component	—
16	Sbarra et al., 2025	Ethiopia (79 zones)	Measles	OR	Dynamic SEIR with MLE + particle filtering	Subnational case notifications, vaccination geodata	2013–2019 (24 age groups)	Reporting rates estimated <3%; vaccine effectiveness ~47% yielded best model fit (AIC)	Steep likelihood surface; suspected cases only; no prospective validation	—
17	Wudu et al., 2024	Ethiopia (East Gojjam)	Measles	OR	ARIMA(3,1,1)	WHO surveillance database	2018–2022 (forecast to 2027)	Model selected via BIC; projected increasing measles incidence through 2027	Single zone; no external validation; no RMSE/MAE reported	—
18	Mollalo et al., 2020	USA	COVID-19	OR	GIS-based spatial modeling (OLS, GWR)	County-level COVID-19 data	2020 (early pandemic)	Demonstrated GIS-based spatial modeling of infectious disease incidence at county level	COVID-19-specific; different transmission dynamics from measles	GIS spatial modeling methodology applicable to measles subnational risk mapping
19	Silva et al., 2024	Portugal	COVID-19	OR	Mobility-based spatiotemporal modeling	Mobility data, COVID-19 case data	Pandemic period	Integrated population mobility data for short-term spatial disease prediction	COVID-19-specific transmission patterns	Mobility data integration methods applicable to modeling

Supplementary Table S1. Characteristics of Studies Included in the Narrative Review

										measles spatial spread
20	Guo et al., 2025	China	Healthcare systems	OR	Interregional population mobility modeling	Interregional mobility data	Not specified	Modeled healthcare resource dynamics based on population movement patterns	Not disease-specific; healthcare resource focus	Population mobility modeling framework applicable to measles outbreak resource planning
21	Hayford et al., 2019	Zambia	Measles/Rubella	OR	Seroepidemiological survey	Post-campaign serosurvey (DBS samples)	2016	Identified rubella immunity gap in 16–30 year-olds (seroprevalence 98.4% vs 89.8% reported coverage); demonstrated added value of nesting serosurveys within coverage evaluations	Single province; cross-sectional design	—
30	Gyebi et al., 2023	Ghana	Measles	OR	ML classifiers (RF, SVM, ANN, NB, DT) vs. GLM	Laboratory-confirmed surveillance data	1,797 suspected cases (78 confirmed positive)	RF best: accuracy 92.11%, sensitivity 0.883, specificity 0.964, PPV 0.963, AUC 0.923; region of residence strongest predictor	Severe class imbalance (random oversampling); only 6 predictor variables; potential performance inflation	—
31	Kujawski et al., 2024	USA	Measles	OR	XGBoost (17 county-level predictors)	County-level demographic, vaccination, mobility data	Tested on 2019 data	Sensitivity 0.72, specificity 0.94, AUC 0.92; logistic regression: sensitivity 0.16, specificity 1.00, AUC 0.91	US-specific; limited PPV; requires high-dimensional geocoded data	—

Supplementary Table S1. Characteristics of Studies Included in the Narrative Review

32	Glock et al., 2021	USA	Measles	OR	ResNet (CNN, transfer learning)	Dermatological images (>1,300)	Not specified	Accuracy 95.2%, sensitivity 81.7%, specificity 97.1%	Controlled dataset; 18.3% false-negative rate problematic for public health; no field validation	—
33	Jiao et al., 2025	Japan	Multiple infectious diseases	OR	LSTM with attention mechanism + mobility data	Epidemic surveillance data, mobility data	Multiple epidemics	LSTM with attention improved spatio-temporal forecasting; gains over baseline ARIMA	Requires consistent mobility data availability	LSTM attention architecture applicable to measles time-series forecasting
34	Chowdhury et al., 2024	Global	Epidemics/Pandemics (general)	R	AI/ML comprehensive review	Literature review	N/A	Reviewed AI/ML methods for epidemic management; identified key deployment barriers in public health settings	Review article; no original analysis	Barrier analysis and deployment frameworks applicable to measles AI tool adoption
35	Cheah et al., 2025	Global	Infectious diseases (general)	R	ML/AI review (surveillance, diagnosis, prognosis)	Literature review	N/A	Reviewed ML applications across infectious disease surveillance, diagnosis, and prognosis	Review article; no original analysis	Surveillance and diagnostic AI frameworks applicable to measles
36	Villanueva-Miranda et al., 2025	Global	Infectious diseases (general)	R	Systematic review (AI in early warning systems)	Systematic literature search	N/A	Documented limited real-world deployment of AI in operational infectious disease surveillance	Systematic review; no original analysis	AI early warning deployment barriers directly relevant to measles surveillance

Supplementary Table S1. Characteristics of Studies Included in the Narrative Review

37	Olawade et al., 2023	Global	Public health (general)	R	Narrative review (AI in public health)	Literature review	N/A	Reviewed AI applications for public health improvement including disease surveillance	Narrative review; no original analysis	Public health AI deployment considerations applicable to measles programs
41	Ru et al., 2023	USA	Measles	OR	Hybrid HDBSCAN/uRF + XGBoost/LR	County-level surveillance and demographic data	Not specified	XGBoost: AUC-ROC 0.920–0.926, AUC-PR 0.522–0.532; LR: higher sensitivity (0.837–0.857) but lower PPV (0.122–0.141)	PPV < 0.37 (>60% false alarm rate); complex pipeline; not externally validated	—
43	Matysiak-Klose et al., 2024	Germany/Europe	Measles	C/D	Epidemiological guidance	Surveillance data (low-incidence settings)	Low-incidence context	Described measles epidemiology, diagnosis, and surveillance challenges in elimination settings	Descriptive; no computational modeling	—
44	Shareef et al., 2024	India	Measles	OR	YOLOv5 (CNN-based detection)	MSID dataset (rash images)	Not specified	92% accuracy, F1 = 0.92 for automated measles rash detection	Single dataset; no clinician comparison; no external validation	—
45	Naik et al., 2025	India	Measles	OR	Hybrid CNN + RF + KNN	Measles lesion images	Not specified (limited dataset)	99% accuracy reported for measles lesion detection	Limited dataset; no external validation; likely overfitting; in-sample performance only	—
47	Cutts et al., 2020	LMICs	Measles	R	Policy models (review)	Literature review	Recent applications in LMICs	Reviewed use of mathematical models for measles control strategy development in LMICs	Review article; no original modeling	—

Supplementary Table S1. Characteristics of Studies Included in the Narrative Review

48	Burtenshaw et al., 2025	USA	Measles	OR	FNN and BINN (DTW feature selection)	Historical outbreak data + CDC estimates	34-week testing period (2025 outbreak)	MSE < 2 (FNN: 1.106; BINN: 1.145); 5-week-ahead predictions aligned with CDC estimates (March 2025)	Preprint; not peer-reviewed; single outbreak context; untested for different epidemic dynamics	—
49	Alemayehu, 2024	Ethiopia	Measles	OR	XGBoost (best of 8 algorithms)	Ethiopian DHS	Children 12–23 months	XGBoost: accuracy 73.9%, AUC 0.813; SHAP identified key dropout predictors (maternal age, education, region)	~26% error rate; cross-sectional data; predictive vs. causal interpretation	—
53	Lara-Benítez et al., 2021	Global (benchmark)	Multiple infectious diseases	OR	DL architecture comparison (8 architectures, >38,000 models)	>50,000 time series across diseases	Various	LSTM most accurate overall; CNN comparable accuracy with better computational efficiency and robustness	General benchmark; may not transfer to sparse, episodic measles data	Architecture comparison directly informative for measles forecasting model selection
54	Eilertson et al., 2019	USA (theoretical)	Measles	OR	Mechanistic model + particle filtering + MLE	Historical measles data	Not specified	Demonstrated particle filtering and MLE for mechanistic model parameter estimation	Methodological focus; limited real-world application scope	—
55	Cheng et al., 2025	Malaysia	COVID-19	OR	Hybrid SEIRV-DNNs (dynamics-informed)	COVID-19 surveillance data	Pandemic period	Demonstrated dynamics-informed neural networks for epidemic trajectory modeling	COVID-19-specific; different R_0 and vaccination dynamics	Hybrid mechanistic–DNN approach applicable to measles scenario modeling

Supplementary Table S1. Characteristics of Studies Included in the Narrative Review

56	Kim et al., 2025	Global	COVID-19	OR	CNN-based prediction (critical evaluation)	COVID-19 health data	Pandemic period	Critical evaluation identifying systematic weaknesses in CNN approaches for epidemic prediction	COVID-19-focused; identified generalizability concerns	Evaluation framework and identified pitfalls applicable to measles CNN applications
57	Aslam et al., 2025	Global	Influenza, mpox, Measles	OR	Continuous Learning (CEL) with EWC	Multi-disease outbreak data	Multiple outbreaks	High R^2; 18% improvement in memory stability over baselines; forgetting rate 65%	65% forgetting rate still substantial for operational use; experimental stage	—
60	Jin et al., 2022	Global	COVID-19	OR	Hybrid ensemble (multiple NNs + reinforcement learning)	COVID-19 surveillance data	Pandemic period	Demonstrated data-driven hybrid ensemble with reinforcement learning for epidemic forecasting	Complex architecture; COVID-19-specific training	Ensemble + reinforcement learning methodology applicable to measles multi-model forecasting
61	Chemkomnerd et al., 2025	Thailand	Healthcare systems (pandemic)	C/D	Scenario-driven simulation	Hospital resource data	Various scenarios	Demonstrated scenario simulation framework for hospital resource management during outbreaks	Healthcare resource focus; not transmission modeling	Scenario simulation approach applicable to measles outbreak resource planning
62	Ristić et al., 2025	Serbia (Vojvodina)	Measles	C/D	Descriptive epidemiological surveillance	Regional surveillance data	Multi-year	Described local measles epidemiology trends and vaccination coverage in regional European context	Descriptive; single region; no computational modeling	—
65	Bozzola et al., 2020	Global	Measles	C/D	Review (digital communication strategies)	Literature review	N/A	Discussed digital communication strategies for measles outbreak risk communication	Conceptual review; no computational modeling	—

Supplementary Table S1. Characteristics of Studies Included in the Narrative Review

68	Cowley et al., 2021	Bangladesh	COVID-19 (SARS-CoV-2)	OR	Genomic + social media + mobile phone data integration	Genomic sequences, social media, mobile phone data	2020	Integrated multiple novel data streams for SARS-CoV-2 lineage mapping and policy support	COVID-19-specific; requires genomic surveillance infrastructure	Multi-source data integration applicable to measles phylogeographic and outbreak attribution analysis

Total: 46 sources (29 original research, 10 reviews, 7 conceptual/descriptive). Among original research: 22 measles-focused + 7 from related diseases.
Non-measles studies highlighted in yellow. Study Type: OR = Original Research; R = Review; C/D = Conceptual/Descriptive.
Transferability Justification provided only for non-measles studies; '—' indicates measles-focused study.
